# Inhalative Nanoparticulate CpG Immunotherapy in Severe Equine Asthma: An Innovative Therapeutic Concept and Potential Animal Model for Human Asthma Treatment

**DOI:** 10.3390/ani12162087

**Published:** 2022-08-16

**Authors:** John Klier, Sebastian Fuchs, Gerhard Winter, Heidrun Gehlen

**Affiliations:** 1 Equine Clinic, Centre for Clinical Veterinary Medicine, Ludwig-Maximilians-University, 85764 Oberschleißheim, Germany; 2 Pharmaceutical Technology and Biopharmaceutics, Faculty of Chemistry and Pharmacy, Ludwig-Maximilians-University, 81377 Munich, Germany; 3 Equine Clinic, Surgery and Radiology, Department of Veterinary Medicine, Free University of Berlin, 14163 Berlin, Germany

**Keywords:** asthma, immunotherapy, allergy, nanoparticle, CpG

## Abstract

**Simple Summary:**

Severe equine asthma is the most common globally widespread non-infectious equine respiratory disease (together with its mild and moderate form), which is associated with exposure to hay dust and mold spores, has certain similarities to human asthma, and continues to represent a therapeutic problem. Immunomodulatory DNA sequences (CpG) bound to nanoparticles were successfully administered by inhalation to severe asthmatic horses in several studies. It was possible to demonstrate a significant, sustained, one-to-eight-week improvement in important clinical parameters: partial oxygen pressure in the blood, quantity and viscosity of tracheal mucus secretion in the airways, and the amount of inflammatory cells in the respiratory tracts of severe asthmatic horses. The immunotherapy with CpG is performed independent of specific allergens. At an immunological level, the treatment leads to decreases in allergic and inflammatory parameters. This innovative therapeutic concept thus opens new perspectives in severe equine asthma treatment and possibly also in human asthma treatment.

**Abstract:**

Severe equine asthma is the most common globally widespread non-infectious equine respiratory disease (together with its mild and moderate form), which is associated with exposure to hay dust and mold spores, has certain similarities to human asthma, and continues to represent a therapeutic problem. Immunomodulatory CpG-ODN, bound to gelatin nanoparticles as a drug delivery system, were successfully administered by inhalation to severe equine asthmatic patients in several studies. It was possible to demonstrate a significant, sustained, and allergen-independent one-to-eight-week improvement in key clinical parameters: the arterial partial pressure of oxygen, the quantity and viscosity of tracheal mucus, and neutrophilic inflammatory cells in the respiratory tracts of the severe equine asthmatic subjects. At the immunological level, an upregulation of the regulatory antiallergic and anti-inflammatory cytokine IL-10 as well as a downregulation of the proallergic IL-4 and proinflammatory IFN-γ in the respiratory tracts of the severe equine asthmatic patients were identified in the treatment groups. CD4^+^ T lymphocytes in the respiratory tracts of the asthmatic horses were demonstrated to downregulate the mRNA expression of Tbet and IL-8. Concentrations of matrix metalloproteinase-2 and -9 and tissue inhibitors of metalloproteinase-2 were significantly decreased directly after the treatment as well as six weeks post-treatment. This innovative therapeutic concept thus opens new perspectives in the treatment of severe equine asthma and possibly also that of human asthma.

## 1. Introduction

The global prevalence and morbidity of human asthma has increased significantly in the last four decades [1]. The prevalence increases by 50% every decade [1]. This has been evident in humans [1]. Severe equine asthma has become the most common non-infectious, chronic, obstructive, inflammatory airway disease in industrialized countries in the northern hemisphere, together with its mild and moderate form, formerly known as inflammatory airway disease (IAD). Severe equine asthma has also been known as recurrent airway obstruction (RAO) (until 2016 when “equine asthma” became the officially accepted term), heaves, chronic obstructive bronchitis/bronchiolitis (COB), and chronic obstructive pulmonary disease (COPD) of the horse (the term is now considered to be obsolete due to pathogenetic differences to human COPD, such as exposure to smoke and minimal reversibility) [2,3,4]. Early descriptions of the clinical presentation of severe equine asthma with the characteristic “heave line” in horses with obstructive pulmonary issues appear in ancient Greek texts by Aristotle from 333 BC [2,5]. In 1656, Markham described heaves in association with housing horses in stables [5]. The first scientific representation in modern times of heaves as an asthma-like syndrome in horses took place in 1964 [2,6].

This overreaction of the airways, which, depending on the reference, has an occurrence in local latitudes between 10 and 20% [7] or maybe even higher [8,9] of adult horses, starting at about 7 years old (reviewed by [2,3,4]) or maybe even earlier [9], with no breed or sex predisposition [2], and possesses certain similarities to human asthma [2,4,10,11]. There are many different human asthma phenotypes, and not all share similarities with severe equine asthma [11]. The most common human asthma phenotypes are allergic (extrinsic) and non-allergic asthma, late-onset asthma, asthma with fixed airflow limitation, and asthma in obese patients [11]. The first three show a good accordance with severe equine asthma [11]. Severe equine asthma has therefore been proposed as an ideal model for the human severe allergic, non-allergic, and late-onset asthma phenotypes [11,12]. Severe equine and human asthma are both characterized by reversible airway obstruction with bronchoconstriction, increased mucus production, airway hyperresponsiveness, and pulmonary remodeling [12]. The pulmonary remodeling features in severe equine asthma closely resemble those of human asthma and make this naturally occurring animal model unique [12]. According to Bond et al. [11], the *allergic phenotype* is characterized by an allergenic trigger (e.g., molds) associated with clinical signs and pathology (increased neutrophil % in BAL and increased respiratory effort at rest), multiple hypersensitivities in some families of horses (insect bite hypersensitivity, urticaria, and increased parasite resistance), and a good response to inhaled corticosteroids. An association exists between IL-4Rα and severe equine asthma, where IL-4Rα upregulates IL-4 expression during disease exacerbation, which promotes isotype switching from IgM to IgE and results in increased IgE in BAL from horses with severe equine asthma. The *non-allergic phenotype* is characterized by a neutrophilic or paucigranulocytic BAL (in severe cases where BAL return is low), chronic innate immune activation, and the chronic activation of peripheral neutrophils, and it often responds less well to inhaled corticosteroids. The *late-onset phenotype* is characterized by decreased baseline pulmonary function during disease exacerbation, occurs in mature/older animals, and can require higher doses of corticosteroids for control [11]. Hulliger et al. [13] revealed a strong similarity on the transcriptomic level between severe equine asthma and severe neutrophilic asthma in humans, potentially through affecting Th17 cell differentiation. This study also showed that several dysregulated miRNAs and mRNAs are involved in airway remodeling [13].

### 1.1. Pathophysiology of Severe Equine Asthma

The pathophysiology of severe equine asthma involves recurring, reversible, cholinergic bronchospasms with air trapping, hyperresponsiveness, or hyperreactivity of the airways, hypercrinia and dyscrinia (dysfunctional sol and gel layer of the physiological airway epithelial secretion) with dysfunctional mucociliary clearance, a decrease in the club/Clara cells (physiological secretory cells), goblet cell metaplasia and hyperplasia, and mucosal edema [2]. A significant luminal migration of primarily neutrophilic granulocytes is observed in the airways as well as submucosal and peribronchial lymphocytic infiltration, partially with mast cells, plasma cells, and eosinophilic granulocytes [2]. This can lead to airway remodeling with hyperplasia and hypertrophy of the smooth muscles and subepithelial and interstitial fibrosis [2]. Clinical signs include cough, exercise intolerance, recurrent seromucosal up to mucopurulent nasal discharge, and depending on the exacerbation phase, severe respiratory distress and significant dyspnea at rest (elevated resting respiratory rate, increased abdominal lift, nasal flaring, intercostal breathing, biphasic exhalation, and protrusion of the anus during exhalation) [2,9,10,11]. On the immunological level, there are conflicting study findings and opinions, so some authors consider the reaction a type I IgE-mediated immediate reaction [14,15,16], while others deem this unlikely since the bronchospasm following allergen exposure is delayed, IgE cannot be regularly demonstrated [17,18], and mast cells usually play a subordinate role [17]. The delayed neutrophilic inflammatory reaction could possibly be indicative of a type III Arthus immune complex reaction [2,19,20]. A cell-mediated delayed type IV immune reaction is also plausible [19], which these authors consider the most likely explanation. According to Fey [9], not further specified nonspecific immune/defense reactions could also play a role. The existence of different phenotypes and endotypes within severe equine asthma, similar to human asthma, is probable [10,11].

Numerous studies on the role of cytokines indicate an excessive expression of proallergic Th2 cytokines in the lungs of asthmatic horses [20,21,22]. Others have additionally demonstrated increased Th1 cytokines in asthmatic horses [23,24], which points to the involvement of the proinflammatory track in the pathogenesis of severe equine asthma and contradicts the possibility of a sole Th2 overreaction. Despite this, it is assumed that severe equine asthma involves a dysfunctional Th1/Th2 balance, with a shift to an excessive Th2 response [25]. Other studies have additionally demonstrated the involvement of the proinflammatory and chemotactic IL-17 from Th17 cells in the pathogenic mechanism of severe equine asthma [26,27]. Transcriptomic data derived from bronchial epithelium (in vivo) stimulated with hay dust extract to identify differentially expressed genes and pathways in severe equine asthma indicate that the most upregulated genes are those involved in immune cell trafficking, neutrophil chemotaxis, immune and inflammatory responses, cell cycle regulation, and apoptosis (reviewed by [11]). The most upregulated chemokine was CXCL13, a B-cell chemoattractant predominantly produced by Th17 (reviewed by [11]). CXCL13 has been shown to be upregulated 8-fold in BALF from human asthmatics compared to controls (reviewed by [11]). The treatment of a sensitized murine asthma model with an anti-CXCL13 antibody reduces inflammatory cell recruitment, bronchial-associated lymphoid tissue formation, and airway inflammation [11].

There is a genetic association between severe equine asthma and microsatellite markers with the IL-4 receptor α-chain (IL-4Rα) gene on equine chromosome 13 [11]. The IL-4Rα gene is associated with the development of asthma, skin allergies, and parasite defense in humans [11]. Severe equine asthma is associated with multiple hypersensitivities, including insect bite hypersensitivity, urticaria, and increased parasite resistance in one high-incidence family [11]. Besides a genetic predisposition with higher familial incidence [28,29,30,31], the high prevalence of severe equine asthma is primarily attributed to the widespread stabling of horses, with permanent exposure to antigens [2,3]. The disease is chronic and is currently not curable (with the exception of the mild and moderate form of equine asthma, previously termed “inflammatory airway disease” (IAD)) [2]. The consistent avoidance of antigens is the foundation for the management of the disease and is essential for sustained therapeutic success in the sense of achieving clinical remission [2,9]. A large number of potentially proinflammatory particles have been identified in stall dust, such as bacterial endotoxins, over 50 different species of mold, peptidoglycans, proteases, microbial toxins, storage mites, organic plant particles, and inorganic dust as well as harmful gases and ammonia that contribute to the detrimental effect [32,33]. A subtype of severe equine asthma, summer pasture-associated severe equine asthma—severe equine pasture asthma (EPA) or its former term summer pasture-associated obstructive pulmonary disease (SPAOPD)—is clinically identical but occurs during the summer months (sometimes also in spring and autumn), as it is caused by seasonal pollen in pastures [3].

### 1.2. Diagnosis of Severe Equine Asthma

The diagnosis of severe equine asthma is made based on anamnesis, thorough clinical examination, and additional tests such as bronchoscopy, bronchoalveolar lavage fluid cytology, and additional tests for the evaluation of lung function and arterial blood gas parameters, if available (reviewed by [4,34]). A reliable evaluation and grading of the disease status of severe equine asthma, including coughing, nasal discharge, respiratory rate at rest and during exercise, and the performance and willingness of the horses to work, can be achieved via the standardized HOARSI questionnaire (Horse Owner Assessed Respiratory Signs Index) with four grades [35]. It is important to exclude other clinically similar diseases of the airways with mainly infectious and rarely vascular, neoplastic, toxic, or metabolic backgrounds. In addition, it is necessary to distinguish the severe form from mild to moderate equine asthma, which can be challenging if severe equine asthma is in partial clinical remission [4]. Mild to moderate equine asthma (formerly termed inflammatory airway disease, IAD) can occur at any age (usually in young and middle-aged horses) [4]. Affected horses have no increased respiratory effort at rest; clinical signs persist for more than 3 weeks, including poor performance and occasional coughing, and often improve spontaneously or with treatment; and recurrence is rare [4]. The diagnosis is confirmed via endoscopy, with excess mucus evident in the tracheobronchial tree (score ≥ 2 with larger but non-confluent blobs for racehorses and ≥3 with confluent or stream-forming mucus for sport/pleasure horses). Regardless of the technique used concerning the instilled volume of fluid, site of sampling, selection of aliquot, and/or sample preparation, BALF cytology values of >10% neutrophils, >5% mast cells, and >5% eosinophils are consistent with mild to moderate equine asthma (references for healthy controls are: neutrophils ≤ 5%, eosinophils ≤ 1%, and metachromatic cells ≤ 2% with 250 mL instilled volume) [4]. Neutrophils above 25% and a mucus score of >2/5 within the trachea are consistent with severe equine asthma [4]. In mild to moderate equine asthma, there is no evidence of airflow limitation based on the esophageal balloon catheter technique (DPmax < 10 cm H_2_O), but with more sensitive methods it is possible to detect airflow limitation and even airway hyperresponsiveness [4]. A moldy hay provocation test can be used in a research setting to discriminate between mild/moderate and severe equine asthma (in remission) based on the development of respiratory effort at rest, but it is not recommended for diagnosis in clinical practice [4]. The analysis of metabolomics in the exhaled breath condensate, such as methanol and ethanol, could offer new diagnostic perspectives in the future for severe equine asthma comparable to those in human medicine (reviewed by [34]).

### 1.3. Treatment Options for Severe Equine Asthma

The cornerstone of the treatment of severe equine asthma is the imperative improvement in stabling conditions with steamed or soaked hay (avoiding dry hay), packaged shavings as bedding (avoiding straw), or permanent outdoor living and the avoidance of potentially triggering antigens, which is, in many cases, very difficult. Additional medical treatment concentrates on improving the clinical symptoms of airway inflammation, bronchoconstriction, and mucus accumulation. The route of administration can be systemic and/or inhalative. Inhalative corticosteroids (ciclesonide, fluticasone, budesonide, and beclomethasone) or systemic corticosteroids (prednisolone and dexamethasone) can be administered to improve clinical signs. In a large, prospective, multicenter, placebo-controlled, double-blinded, clinical trial with 224 horses with severe equine asthma, using a soft mist inhaler and a ten-day (5 d twice daily and 5 d once daily) inhalation treatment, Pirie et al. [36] demonstrated that ciclesonide is efficacious in the treatment of severe equine asthma, with at least a 30% or greater reduction in the weighted clinical score of 73% of the horses in the ciclesonide group and 43% of the horses in the placebo group. The reduction in the mean weighted clinical score (severe: 15–23 points; moderate: 11–15 points; mild: 5–10 points) after 10 days of treatment was 7.2 ± 4.8 in the ciclesonide-treated group, compared to 3.8 ± 4.4 in the placebo group. The reduction in the weighted clinical score (82% sensitivity and 70% specificity [37]) after ciclesonide administration was greater in horses with severe clinical signs compared with horses with moderate clinical signs [36]. Owners recognized an improved quality of life after 10 days of treatment in 69% of ciclesonide-treated horses, compared to 43% of placebo-treated horses [36]. Few systemic and local adverse events of ciclesonide were observed [36]. No lung function testing or lower airway cytology of BALF or mucus scoring via endoscopy were performed in this study [36]. Lavoie et al. [37] showed that dexamethasone per os (0.066 mg/kg once daily for 14 d) and inhalative ciclesonide (twice daily for 14 d) significantly improved lung function, and dexamethasone was superior to ciclesonide. The effect was lost 7 days post-treatment for both [37]. Usually, clinical signs reappear quickly after treatment cessation if the environment is not improved concurrently [38]. Beclomethasone [39,40,41] and fluticasone [42,43,44,45] have been shown to be efficacious in controlling airway obstruction in severe equine asthma, although the magnitude of the response was higher with systemically administered dexamethasone than with inhaled beclomethasone [46]. While improving clinical signs and lung function, corticosteroids have generally been found to be only mildly to not effective in controlling the neutrophilic inflammation in equine asthma (reviewed by [38,40,47,48]), unless combined with antigen avoidance [24,45]. The poor response of neutrophilic inflammation to corticosteroids is not limited to horses, as similar findings have also been reported in human patients with neutrophilic asthma [49]. The improvement in lung function via ciclesonide was lost one week after the discontinuation of the therapy, but the weighted clinical score improvement remained significant up to one week post-treatment [36].

Bronchoconstriction can be improved medically via systemic bronchodilators (clenbuterol and butylscopolamine) or inhalative bronchodilators (salbutamol, salmeterol, and ipratropium bromide) [2,3,4,50]. Finally, expectorants (dembrexine and acetylcysteine) or inhaled saline (isotonic or hypertonic) can be administered to improve the liquefaction and transport of mucus, and mild exercise can also be beneficial. Expectorants are certainly less important than the aforementioned strategies in controlling inflammation and bronchoconstriction. Allergen-specific immunotherapy could potentially become an interesting and promising therapy approach in the future for some cases of allergic severe equine asthma. However, to the best of the authors’ knowledge, the current scientific literature scarcely includes any reports of allergen-specific immunotherapy as a successful treatment option for severe equine asthma, likely due to the multifactorial nature, the different phenotypes of the disease, and the diagnostic limitations of the currently commercially available allergy tests. The question arises whether it is possible to affect the pathophysiology on the immunological level and prevent or reduce the occurrence of a hypersensitivity reaction.

### 1.4. Th1/Th2 Balance

The Th1/Th2 balance in the body is vital to the homeostasis of the immune response [51], and a shift towards an excessive Th2 response can result in allergic disease [51,52]. Physiologically, Th2 cells play an essential role in fighting extracellular parasites such as intestinal helminths [53]. This path is oriented towards the mucosa of the gastrointestinal and respiratory tracts, where contact with the external world occurs [53]. These surfaces are predisposed to the parasitic invasions against which IgE defends [53]. Antigen-presenting cells (APCs) at these barriers to the external world are programmed to the Th2 response [53]. Natural infections by bacteria and viruses result in a cell-mediated proinflammatory Th1 immune response by the immune system [51]. In contrast, an allergy-favoring humoral Th2 response is dominant in the neonatal immune system [51]. It is suspected that the decline of infectious diseases during the immune system’s early phase of development could be a cause of the increased incidence of allergies [54,55,56]. Contact with microbial pathogens during this developmental phase appears to have a significant effect on the imprinting of the immune system [54,56]. Certain studies have shown that children who grow up on farms and have regular contact with animals develop fewer allergies than children in cities [54,56]. The interaction between the environment, the microbiome, and the immune system is probably far more complicated. The number of recent studies analyzing the microbiome of the gut and lung of healthy and severe asthma-affected horses is increasing, offering new insights and perspectives in the treatment and understanding of the pathophysiology of severe equine asthma [57,58]. The immunological interaction between the gut and lungs, termed the gut–lung axis, has been intensely studied in humans and provides new insights into how metabolites produced in the gut influence the immune system in the lungs (reviewed by [34]).

### 1.5. Toll-Like Receptors

The intracellular recognition of the CpG motif occurs via toll-like receptor 9 (TLR9), one of the most important pathogen recognition receptors of the innate immune system [59] and an evolutionarily highly conserved type I transmembrane protein [60]. To date, ten different TLR classes are known in humans, and thirteen are known in vertebrates [61]. Due to individual ligand specificity, each receptor class only recognizes certain binding partners. TLRs can differentiate between “endogenous” and “foreign” and only react to a pathogen-associated molecular pattern (PAMP) during an infection [62,63]. The majority of TLRs (TLR1, TLR2, TLR4, TLR5, TLR6, and TLR11) are located on the cell surface [64], while TLR3, TLR7, TLR8, and TLR9 are localized intracellularly within endosomes [62]. The ligand binding here occurs in the acidic environment of the endosomes, which is a prerequisite for the cellular activity, the dimerization of the TLRs, and their stabilization [63]. TLR9 is specialized to single-stranded CpG DNA [63]. In equine lungs, Schneberger et al. [65] could identify TLR9 in intravascular macrophages, alveolar macrophages, bronchial epithelial cells, capillary endothelial cells of the lungs, type II epithelial cells of the alveolar septa, and neutrophilic granulocytes. Via lipopolysaccharide (LPS) treatment, the level of TLR9 expression as well as the number of TLR9-positive cells could be significantly increased [65]. Cytosine methylation, as it most commonly exists in the mammal genome, or an inversion of the cytosine–guanine dinucleotides (GC) inhibit TLR9 activation [59]. The differentiation between endogenous and foreign DNA is dependent both on the low CpG content and the high rate of cytosine methylation [66] as well as on the protection of the endosomal localization of TLR9 against constant activation by endogenous DNA [67,68].

The nucleotide sugar backbone is of particular importance for TLR9 activation [63]. The synthetic ODN possesses a modified sugar backbone (phosphorothioate), in contrast to the naturally occurring ODN (phosphodiester) [63], and therefore possesses a receptor affinity 100 times stronger than the natural phosphodiester [63].

### 1.6. CpG-ODN

Unmethylated cytosine–phosphate–guanine oligodeoxynucleotides (CpG-ODN) occur primarily in prokaryotic DNA and stimulate the eukaryotic immune system [51,64,69,70]. In mammalian DNA these motifs are quite rare (suppressed) and they are usually methylated (shut down) [70]. These CpG-ODN motifs are able to downregulate excessive allergic immune reactions [51,64,69,70,71]. The use of synthetic CpG-ODN mimics the effect of a bacterial or viral infection and makes use of the body’s own immune system. Thus, CpG-ODN represents a large therapeutic potential for allergic diseases [64]. Gelatin nanoparticles (GNP), as a molecular transport system, protect CpG-ODN from premature degradation by ubiquitous nucleases and simultaneously improve the cellular absorption of the DNA molecules in the target cells of the immune system [72]. First, a CpG motif effective for use in equine bronchoalveolar cells (BAL cells) was identified in an in vitro study on the basis of existing sequence-dependent species specificity [73,74]. Second, the extent to which a specific immunomodulatory effect of the administered CpG-ODN can be demonstrated in equine BAL cells was determined [73,74]. The ability of GNP to serve as an effective molecular transport system for CpG-ODN in equine BAL cells was confirmed [74]. The previously identified CpG-ODN sequence was administered in subsequent in vivo studies to horses with severe equine asthma and was thereby examined for its local and systemic tolerability and its therapeutic effect on clinical and immunological parameters [75,76,77,78,79].

### 1.7. Immunology of CpG-ODN

**Figure 1:**
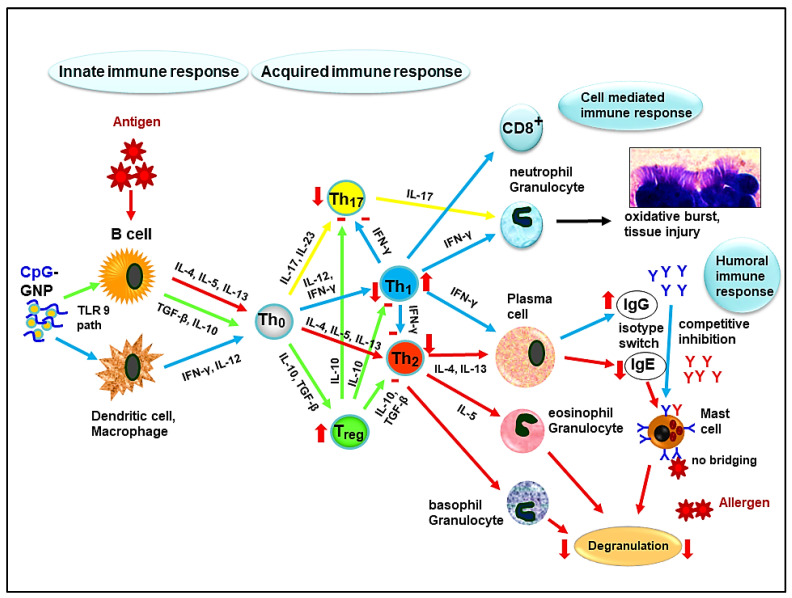
Schematic drawing of the CpG-induced immunological mechanism. The influence of CpG-GNP on the innate and acquired immune system is depicted with particular consideration of the T-helper-cell subsets. After recognition by dendritic cells and macrophages or B lymphocytes upon antigen contact, naive Th0 cells differentiate into Th1, Th2, Th17, or Treg in the appropriate cytokine patterns (Th1 lineage: IL-12 and IFN-γ; Th2 lineage: IL-4, IL-5, and IL-13; Treg lineage: IL-10 and TGF-β; Th17 lineage: IL-17 and IL-23). Depending on the path taken, stimulation (arrow up) or inhibition (arrow down or minus sign) results. This influences immunoglobulin class switching (to IgG or IgE) by plasma cells, the competitive inhibition of IgE by IgG on mast cells, the prevention of cross-linking upon recurrent allergen contact, and no degranulation of the effector cells (mast cells, eosinophils, and basophils). The regulating influence on the oxidative burst and tissue injury in the respiratory tract is caused by migrating neutrophils through the activation of Treg and the regulation of excessive Th1 and Th17 immune responses. (Figure modified according to [73]).

Natural bacterial and viral infections train the immune system toward a cell-mediated proinflammatory Th1 immune response [51]. The contact with microbial pathogens, or synthetic CpG-ODN, leads to the differentiation of naive CD4^+^ T-helper cells into a specialized subclass of Th1 lymphocytes (Figure 1) [70]. These, in turn, release the proinflammatory cytokines IL-12 and IL-18, which mediate an increase in IFN-γ [70]. This central Th1 cytokine has an inhibitory effect on allergy-mediating Th2 cytokines such as IL-4, IL-5, and IL-13 (Figure 1) [64]. This differentiation in favor of Th1 cells (Th1 shift) is largely dependent on the synthesis of IL-12 [70]. Of particular importance in this immunologic event is the activation of regulatory T cells (Treg) by CpG-ODN [80,81]. This leads to the production of IL-10, which causes a peripheral T-cell tolerance, possesses anti-inflammatory as well as antiallergic effects [82], and inhibits Th1 cytokines (IFN-γ) as well as Th2 cytokines (IL-4) [83]. It is therefore of particular interest in the study of excessive allergic and inflammatory diseases such as severe equine asthma. The CpG sequences activate the transcription of the cells within 15 min [84], inducing the production of T-helper 1 (Th1) cytokines by antigen-presenting cells (APC) (Figure 1) [84]. In addition, this causes an increased expression of the major histocompatibility complex (MHC I and II) and costimulatory molecules by APC [69]. T cells can only recognize antigens in their processed form in the presence of endogenous molecules (MHC restriction). If, upon contact with an antigen, CD4^+^ T-helper cells are subjected to a cytokine milieu of IL-4 by APC, the Th2 lineage is stimulated, which as a result, leads to IgE production by the activated B lymphocytes [85]. Furthermore, B-cell activation, independent of T cells, occurs through toll-like receptor 9 (TLR9) [64]. The B lymphocytes, as part of the APC, differentiate themselves in short-lived plasma and memory cells. The memory cells, upon new contact with antigens, increase the production of antibodies by eight to ten times [53].

### 1.8. Molecular Signal Transduction of the CpG-ODN

**Figure 2:**
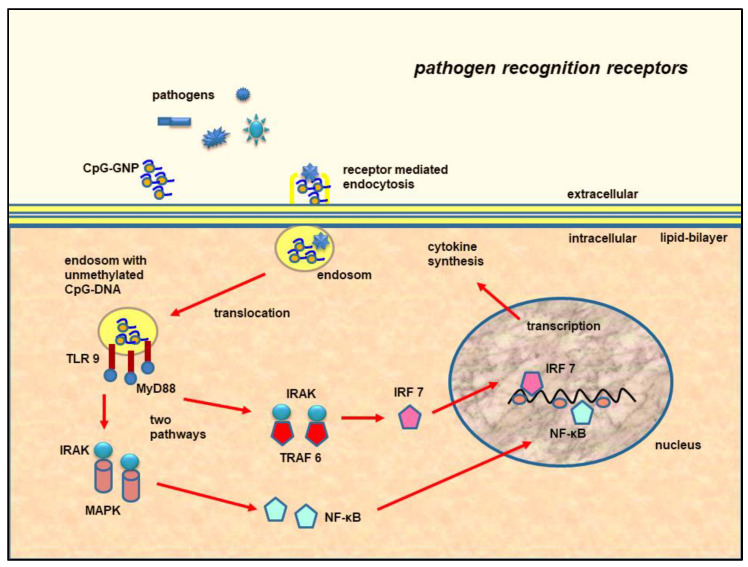
Schematic drawing of TLR9-mediated signal transduction and the intracellular uptake of the pathogens or CpG-GNP over receptor-mediated endocytosis and translocation in endosomes. Recognition by pathogen recognition receptor TLR9 (toll-like receptor 9) triggers a signal cascade over MyD88 (myeloid differentiation primary response gene 88), IRAK (IL-1-receptor-associate kinase), TRAF 6 (TNF receptor-associated factor 6), and IRF 7 (interferon-regulatory factor 7) or IRAK and MAPK (mitogen-activated protein kinase), with the resulting activation of transcription factor NF-κB (nuclear factor-κB). The induction of the transcription of genes encoding specific cytokines leads to the subsequent protein biosynthesis of the cytokines. (Figure modified according to [73]).

CpG-ODN are taken up via endocytosis and transported to the endosomal compartments of the cells through acidic vesicles (Figure 2) [64,71]. The intracellular signal transduction of the CpG-ODN occurs through a dimerization of the receptor molecules [71] with an allosteric conformational change in the cytoplasmic domain that leads to the recruitment of signal adaptor molecules (MyD88) and signal transduction molecules such as the IL-1 receptor-associated kinase (IRAK) and the mitogen-activated protein kinase (MAPK) as well as IFN regulatory factors (Figure 2) [71]. This signal cascade leads to the activation of the transcription factor nuclear factor-κB (NF-κB), with subsequent cytokine production (Figure 2) [71]. One of the central transcription factors of the CpG effect is Tbet (Th1-specific T-box transcription factor) [71]. This inhibits the Th2-associated antibody isotypes and increases the Th1-associated antibody isotypes [71]. The regulatory cytokine IL-10, in a negative feedback loop through the upregulation of CpG activity, leads to a downregulation of the CpG effects [71].

### 1.9. Nanoparticulate Transport Systems of CpG-ODN

CpG-ODN can be successfully transferred to target cells with the aid of various delivery systems [72,86]. This improves the cellular uptake of the CpG-ODN, while steric shielding protects the CpG-ODN molecules from premature degradation by endogenous nucleases and results in a sustained enhancement of the immunostimulatory effect of the CpG-ODN [72]. However, free CpG-ODN display problems in their cellular uptake, stability, and specificity for target cells [72]. When packaged in nanoparticulate delivery systems, this leads to a local lymphatic uptake without systemic circulation [72,87,88] and increases the local rate of phagocytosis, through which CpG-ODN reaches TLR9 in the endosomes directly [87]. The CpG-ODN A class consists of a central palindromic sequence and poly guanine tails on both ends as well as a mixed phosphorothioate and phosphodiester sugar backbone, contrasting with the CpG-ODN B class’s pure phosphorothioate backbone and the C class’s mixture of both classes with an effect on IFN-α, IL-6, and IgM production [89]. The CpG-ODN A class is quickly degraded in vivo by ubiquitous DNases but can be protected against degradation by steric shielding on gelatin nanoparticles or by packaging it in virus-like particles (VLP) [86]. Unpackaged CpG-ODN, on the other hand, spread throughout the organism, and under certain circumstances can lead to shock reactions or splenomegaly [90,91]. Lipid nanoparticles have been proven to be an effective delivery system for CpG-ODN [86]. The packaging of CpG-ODN in nanoparticles leads to enhanced effectiveness, a longer life span, a passive accumulation at the site of the disease incidence, and a reservoir effect of the CpG-ODN with delayed release [86]. Colloid particle formulations move in the same proportions as microorganisms and are therefore better phagocytosed through corresponding defense cells [72].

### 1.10. Gelatin Nanoparticles

Zwiorek et al. [72] established a new delivery system with gelatin nanoparticles (GNP) out of type A porcine gelatin (175 Bloom) combined with cholamine with a pH-independent cationic surface via a quaternary amino group. The loading of the cationic GNP with negatively charged CpG-ODN occurs through electrostatic attraction. The optimal load of GNP is a ratio of 1:20 (5%). At higher loads, the GNP may lose their stability and display a tendency to aggregate, most likely due to their neutral surfaces [72]. The gelatin nanoparticles themselves are immunologically inert, which is a condition for repeated application in the organism and conjugation with immunomodulatory substances [72]. GNP polymers display excellent biodegradability and very good biological compatibility [72,88,92]. The functional groups on the nanoparticles can be conjugated with ligands, making them suitable as a delivery system for pharmacological substances [72].

The aerodynamic stability of GNP after nebulization has been proven [92,93]. Fuchs et al. [94] extensively investigated various nebulizer systems, the appropriate measurements of the particle sizes after nebulization, and other studies on the alveolar mobility of the nebulized particles and the in vitro cytokine expression of nebulized particles in cell cultures of equine BAL cells. With an active vibrating mesh nebulizer (AeroNeb Go micropump nebulizer, Aerogen, Galway, Ireland) the second-stage particle deposition in an impinger was up to 65.68 ± 11.2% of the nebulized dose [94]. The higher the viscosity of the dispersion and the lower the surface tension of the particle-comprising droplets to be nebulized, the smaller the nebulized particles become [40,94]. This is of great significance for alveolar mobility (1–5 µm particle size window). The ideal load has been determined to be 30 µg CpG-ODN per 600 µg GNP (5%) [72]. Phagocytosis has been identified as the main uptake mechanism in the target cells. Zwiorek et al. [72] could demonstrate in vitro that positively charged particles, in contrast to neutral and negatively charged formulations, are better phagocytosed through DC and macrophages. GNP-bound CpG-ODN stimulate a cytokine response that is two to three times greater than the response to unbound CpG-ODN [72]. CpG-GNP leads to an upregulation of the expression of MCH II and CD86 surface molecules [72]. CpG-GNP increases the immunogenicity of additionally transported antigens and enhances the activation and imprinting of T cells by DC [72]. This is significant for a combined application with potential allergens in specific immunotherapy (SIT). The phagocytosis of larger particles (>200 nm) results in a longer retention in endosomal vesicles and positively influences the induction of cytokine synthesis [72]. Unloaded GNP displayed no induction of cytokine expression, meaning that they were immunologically inert. Particles with greater diameters (300 nm) were superior to the smaller particles (150 nm) in reference to cytokine induction (IL-12 production was three times greater) [72]. At no point in vitro or in vivo were any toxic effects observed. None of the mice treated with CpG-ODN/GNP displayed detectable levels of antibodies against GNP after three weeks. Zwiorek et al. [72] therefore concluded that no direct immune response to the protein matrix of the delivery system occurs.

### 1.11. CpG-ODN as an Adjuvant

Many commercial vaccines have used aluminum hydroxide (alum) as an adjuvant for decades. It, however, blocks the activation of CD8^+^ cytotoxic T lymphocytes [84]. Most adjuvants only enhance the Th2 response without stimulating the cellular immune response, with the exception of the *Bacillus Calmette-Guérin* (BCG) adjuvant, which also activates the Th1 lineage [84]. CpG-ODN activate both the humoral as well as the cell-mediated immune responses [95]. In contrast to other adjuvants in vaccines, such as Freund’s adjuvant, CpG-ODN display no sterile abscess formation, regardless of the administration route [84]. Ziegler and colleagues [96] compared two TLR agonists, monophosphoryl lipid A (MPLA) and a C-class CpG-ODN, in vitro with equine PBMCs from healthy and insect bite hypersensitivity (IBH)-affected horses. MPLA induced IL-10 secretion in all horses, with and without *Culicoides* allergens, while suppressing the antigen-induced production of IFN-γ, IL-4, and IL-17. CpG-ODN significantly increased IFN-α, IFN-γ, and IL-4 production. MPLA was seen as a promising adjuvant candidate for allergen-specific immunotherapy (ASIT) in horses, while C-class CpG-ODN was considered to be an unsuitable adjuvant for ASIT because of the induction of IFN-γ and IFN-α and thus may be a useful adjuvant in combination with vaccines for equine infectious or neoplastic diseases [96]. Ziegler et al. [97] showed in vitro that the addition of an adjuvant (MPLA or CpG-ODN) to equine PBMCs obtained from healthy and IBH-affected horses further enhanced the effect of dendritic cell-binding peptides by significantly increasing the production of IFN-γ, IL-4, IL-10, and IFN-α (CpG-ODN) and IL-10 (MPLA) while simultaneously suppressing IFN-γ, IL-4, and IL-17 production (MPLA). The combination with MPLA seems to be a promising option for improving ASIT efficacy in horses, while the combination with CpG-ODN increases the effector immune response to recombinant antigens [97].

Nearly all vaccine studies on CpG-ODN to date have used B classes as adjuvants due to their specific stimulatory effect on B lymphocytes [71]. The synergistic effects between TLR9 and B-cell receptors result in an activation of the humoral immune response, which leads to the stimulation of antigen-specific B cells, inhibits B-cell apoptosis, and increases the survival rate and IgG class switching [71]. No other vaccine adjuvant effects a Th1 immune response as strong as that of CpG-ODN [71]. The mucosal administration activates local and systemic humoral and cellular immune responses and thus imparts superior protection against infection [71]. Vaccine studies have demonstrated that the low protective antibody concentrations after vaccination in HIV infections can be significantly increased through the combination with CpG-B (CpG 7909) [71]. Some RNA viruses display CpG suppression in their genomes and thus evade recognition by the immune system [98]. This is the case with HIV, which displays clear CpG suppression [98]. The introduction of CpG motifs in the HIV genome demonstrated a clear inhibition of the gene expression of the human immunodeficiency virus [98].

In the vaccination against hepatitis B, the seroprotective antibody level with the combined CpG-ODN B administration increased dramatically (100%) and more quickly and was also sustained for a longer period of time (over 3.5 years) (reviewed by [71]). In an anthrax vaccine study, CpG-ODN reached the toxin neutralization level at half the number of days (22 days) in comparison to the control group (including an antibody titer with an 8× increase) (reviewed by [71]). In mice, vaccine doses could be decreased by up to 99% in combination with a CpG-ODN adjuvant [71]. A reduction in the vaccine dose is especially relevant to influenza vaccines in order to enable adequate production of the vaccines [71]. A tenth of the normal influenza vaccine dose, in combination with CpG-ODN, is sufficient to obtain an equal antigen-specific IFN-γ level (reviewed by [71]). The prophylactic administration of CpG-ODN in mice led to transient protection against a wide range of viral, bacterial, and parasitic pathogens such as *Bacillus anthracis*, *Listeria monocytogenes*, the Ebola virus, and the vaccinia virus (reviewed by [71]). Depending on the route of administration (oral, inhalative, or by injection), this protection upon one-time administration of CpG-ODN ranges from one day to two weeks [71].

The direct combination of CpG-ODN with certain allergens leads to an antigen-specific Th1 immune response and simultaneously to the suppression of Th2-mediated allergic asthma [89]. The combination with allergens as a specific immunotherapy is used to combat the major human allergen *Ambrosia artemisiifolia* [61]. A conjugation of the CpG-ODN to specific antigens enhances the uptake of antigens and reduces the necessary antigen quantity [71].

In the horse, CpG-ODN has been administered as a vaccine adjuvant for equine influenza and *Rhodococcus* vaccines [99,100]. Furthermore, CpG-ODN has been successfully administered in vivo in a pilot study on canine atopic dermatitis in combination with liposomes and specific allergens, resulting in a significant improvement in the pruritus [101] and in a study on canine atopic dermatitis without the addition of allergens with gelatin nanoparticles as the drug delivery system [102]. In both studies, the CpG-GNP treatment resulted in a significant decrease in IL-4 mRNA expression in the blood [101,102]. The clinical improvement was determined to be similar to that of specific immunotherapy [102]. An in vivo study on allergic rhinitis and asthma (phase I and IIa) in humans demonstrated the tolerability and clinical effectiveness of CpG-ODN administered in combination with allergens [89]. CpG-ODN have also been used in human tumor therapy. Several clinical studies reviewed by Adamus and Kortylewski [103] with TLR9 agonists as a monotherapy have demonstrated the very good tolerability and security of CpG-ODN.

### 1.12. Inhalative Nanoparticulate CpG Immunotherapy of Horses with Severe Equine Asthma

Because no other comparable studies on this specific subject have been performed by other groups, the authors must note here that independent replication was not possible, and the risk of author bias in the available data cannot be excluded.

#### 1.12.1. In Vitro Trials

The goal of an in vitro study on inhalative nanoparticulate CpG immunotherapy was to identify the optimal stimulatory CpG sequence for horses in consideration of the species specificity of the CpG motifs in equine bronchoalveolar lavage (BAL) cells with regard to an immunomodulatory effect (Th2/Th1 shift) [74]. Gelatin nanoparticles (GNP) were used as the drug delivery system. BAL cells were obtained from horses with severe equine asthma and healthy horses and subsequently incubated with five different CpG-ODN sequences (classes A, B, and C) and an ODN sequence without a CpG motif. The cytokine release of IL-4, IL-10, and IFN-γ was then determined via quantitative equine capture ELISA (R&D systems, Minneapolis, MN, USA) in order to detect an allergy-mediated Th2 immune response (IL-4) and/or a proinflammatory Th1 response (IFN-γ). Due to its specific anti-inflammatory and antiallergic effects, IL-10 was considered a positive regulatory cytokine in the pathophysiology of severe equine asthma. The results in the asthmatic horses revealed both a significant upregulation of IL-10 and IFN-γ as well as a downregulation of IL-4 [74]. The cell cultures of the healthy horses had a significantly greater cytokine release in response to the administered stimuli in contrast to the cell cultures of the horses with severe equine asthma. In the comparison of all five CpG sequences, the A class 2216 displayed the strongest immunomodulatory effect on equine BALF cells [74], and for that reason, it was selected for the follow-up clinical studies.

#### 1.12.2. In Vivo Trials

**Figure 3:**
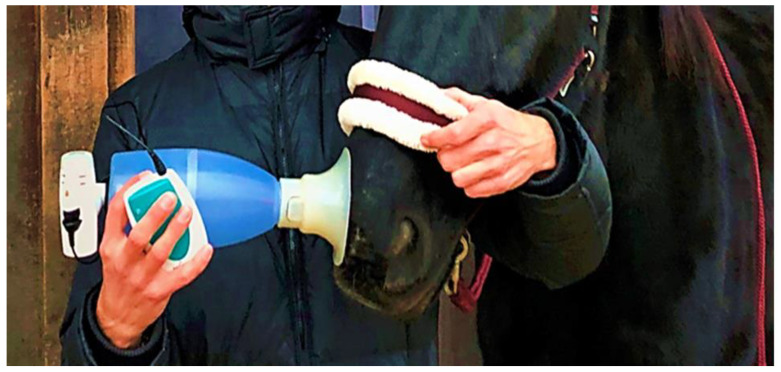
Inhalation device. Combination of “AeroNeb Go micropump nebulizer” (Aerogen, Galway, Ireland) and “equine haler” (Equine HealthCare Aps, Hoersholm, Denmark) for in vivo inhalation studies on horses.

In the following in vivo trial, the previously identified sequence was administered via inhalation to healthy and asthmatic horses [73,75]. Without altering external environmental factors, a significant improvement in the examined parameters (neutrophil percentage in tracheobronchial secretion (TBS), arterial oxygen pressure, tracheal mucus, and respiratory rate) was evident after three and five inhalations. However, the percentage of neutrophils within the trachea does not correlate well in all cases with the percentage of neutrophils within the lungs gained via BAL. Consequently, the reduction in neutrophils within the trachea does not necessarily allow a direct conclusion regarding the neutrophils within the lungs. The inhalations were administered every second day (once daily for ca. 10 min) (Figure 3).

In addition, the BAL cells obtained after inhalation displayed a significantly higher stimulation potential (increased IL-10 release and decreased IL-4 release in vitro in contrast to before the inhalation) [73,75]. Consequently, it was concluded that the inhalation treatment leads to an alteration of the cell population in vivo and results in the upregulation of Treg cells (increased IL-10 production). The upregulation of IL-10 and IFN-γ in the BAL could also be determined in vivo after the inhalation treatment [75].

The clinical and immunological parameters of the GNP-bound CpG-ODN formulations were examined further in the subsequent double-blind, placebo-controlled, and prospective randomized clinical phase I and IIa field study [76]. In the study, 24 horses with severe equine asthma received inhalative treatment (verum group n = 16; placebo group n = 8) five times with an interval of two days and were examined before and immediately after treatment cessation as well as four weeks after the treatment’s conclusion. The CpG-GNP treatment achieved a 4-week persistent and significant improvement in 70% of the examined parameters, including breathing type, auscultation, alveolar–arterial oxygen gradient (AaDO_2_) (calculated according to the current atmospheric pressure and the measured blood gas values PaO_2_ and PaCO_2_ (AaDO_2_ = (atmospheric pressure − 47 mmHg) × 0.2095 − PaCO_2_ − PaO_2_)) [78], the neutrophil percentage in the tracheobronchial secretion, and the amount and viscosity of tracheal mucus as well as the nasal discharge of the horses in their accustomed environmental conditions with sustained exposure to allergens [76]. The positive results of this exploratory field study revealed new possibilities beyond the conventional symptomatic treatments and could therefore also serve as a potential therapy model for human asthma.

A further study investigated whether two additional specific allergens in the sense of an allergen-specific immunotherapy (ASIT) (according to the results of a functional in vitro test on each horse) could enhance the immunomodulatory capacity of the CpG-GNP formulation in horses with severe equine asthma both in regards to a strengthened immunological response as well as a longer sustained effect in contrast to monotherapy with CpG-ODN [78]. Furthermore, the study investigated whether a longer inhalation therapy (seven inhalations in comparison to five) could achieve a superior therapy effect that was sustained longer or was stronger in comparison to the earlier study [76]. Twenty horses with severe equine asthma were divided into two groups. Based on a functional in vitro test, eleven horses were administered two specific allergens (Artu Biologicals, Europe B.V., Lelystad, the Netherlands) in increasing concentrations (beginning with 0.6 mL and increasing to 1.2 mL) every second day for a total of seven administrations in addition to CpG-GNP. The treatment with solely CpG-GNP (n = 9 severe equine asthma horses), as well as in combination with relevant allergens (n = 11), resulted in no significant improvement in the parameters of respiratory rate, breathing type, nasal discharge, and the quantity and viscosity of tracheal mucus six weeks after the inhalation treatment [78]. There were no significant differences between the two treatment groups in either the clinical parameters or the cytokine profiles in tracheal wash sampling (IL-10, IFN-γ, and IL-17). The IL-4 concentrations decreased significantly in both groups. An allergen-independent CpG-GNP immunotherapy therefore has great potential as a treatment for equine and possibly also human asthma. An additional allergen component did not achieve any significant advantage.

In severe equine asthma, increased matrix metalloproteinase (MMP) expression contributes to pathological pulmonary tissue damage, while tissue inhibitors of metalloproteinases (TIMPs) combat MMP overexpression and pulmonary fibrosis (reviewed by [79]). Barton et al. [79] showed that CpG-GNP inhalation presents a possible effective therapy that can work against the imbalance in MMP and TIMP expression in pulmonary tissue in severe equine asthma. Matrix metalloproteinases (MMP-2/MMP-9) and tissue inhibitors of metalloproteinase (TIMP-1/TIMP-2) concentrations were determined in tracheal wash sampling by equine ELISAs before and two and six weeks after CpG-GNP inhalation [79]. MMP-2, MMP-9, and TIMP-2 concentrations were significantly decreased directly after the treatment as well as six weeks post-treatment [79]. The imbalance in the elastolytic activity appears to be improved by the CpG-GNP inhalation for at least six weeks after treatment, which could possibly reduce the remodeling of the extracellular matrix [79]. The CpG-GNP inhalation could therefore represent an effective therapy for the prevention of pulmonary fibrosis in severe equine asthma [79].

Finally, a prospective, randomized, double-blind clinical field study examined 29 horses with severe equine asthma to explore the dose-dependent effect (the single dose of 187 µg CpG from the previous studies and the double dose of 374 µg CpG) of the inhalative immunotherapy with CpG-GNP, a repeated inhalation treatment in the sense of a booster effect (10 inhalations every second day in comparison to 5 and 7 in the former studies), and a later follow-up examination after a period of 8 weeks (in comparison to 4 and 6 weeks) without further treatment or changes in environmental factors [77]. Here, the therapy concept was compared with a traditional inhalative treatment with beclomethasone (once-daily inhalation with 1600 µg beclomethasone over 10 days). No significant difference could be found between the two CpG-GNP doses (single and double doses) [77]. With regard to a sustained effect over 8 weeks, the CpG-GNP treatment proved advantageous compared to the beclomethasone inhalation in the parameters of respiratory rate, the quantity and viscosity of tracheal secretion, and neutrophils in the BAL [77]. The single-dose CpG-GNP resulted in a significant improvement in 82% of the examined parameters, while the double-dose CpG-GNP resulted in a significant improvement in 72% of the parameters examined directly after the inhalation regimen [77]. With regard to a persistent effect over 8 weeks, the single-dose CpG-GNP showed a significant improvement in 100% of the parameters in comparison to the initial values, and the double-dose CpG-GNP showed a significant improvement in 67% of the parameters [77]. Regarding the immunological parameters in the bronchoalveolar lavage, significant decreases in IL-4 and IFN-γ were evident with the single-dose CpG-GNP treatment [77]. CD4^+^ T lymphocytes gained via BAL were demonstrated to significantly downregulate mRNA expression (realtime PCR) of Tbet and IL-8 8 weeks after CpG-GNP administration in comparison to baseline values [77]. The double dose did not present any advantage in comparison to the original single dose. On the immunological level, an anti-inflammatory and immunomodulatory effect away from a Th2-dominated immune response was detected.

**Figure 4:**
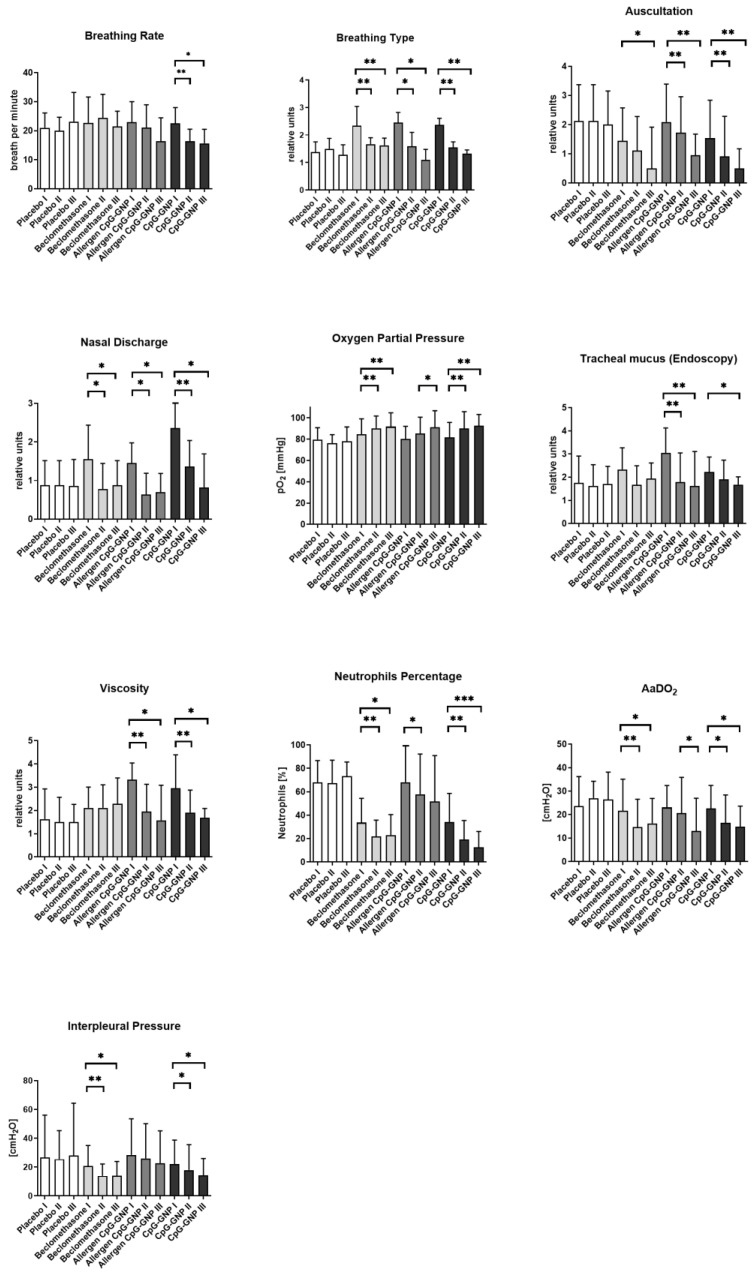
Comparison of the results of the three studies with placebo, beclomethasone, allergen and CpG-GNP, and CpG-GNP inhalation in reference to clinical, endoscopic, cytological, and laboratory parameters: Calculated values (means ± SDs) of *p* < 0.05 represented as *; *p* < 0.001 as **; *p* < 0.0001 as ***. Increase or decrease in respiratory rate, breathing type, nasal flaring, auscultatory findings of the lungs, indirect measurement of pulmonary pressure, partial oxygen pressure, AaDO_2_, neutrophilic granulocytes in the BAL, nasal discharge, quantity and viscosity of secretion in the endoscopy between first (I), second (II), and third (III) examination with placebo (white bar, *n* = 8 severe equine asthma-affected horses; GNP and highly purified water; five inhalations, 48 h dosing interval), beclomethasone (light gray bar, *n* = 9 severe equine asthma-affected horses, seven inhalations, 48 h dosing interval, CpG-GNP with two specific allergens chosen according to an allergy test), and CpG-GNP (dark gray bar, *n* = 11 severe equine asthma-affected horses, 10 inhalations, 48 h dosing interval, normal single dose of 187 ug CpG). The time of the third examination (follow-up without further treatment or improvements in stabling during this time) was dependent on the respective study: with placebo, after 4 weeks [76]; allergen and CpG-GNP after 6 weeks [78]; CpG-GNP after 8 weeks [77].

The referenced studies investigated the first inhalative nanoparticulate equine immunotherapy (Figure 4) [73,75,76,77,78,79]. Beyond the conventional therapy approaches through symptomatic treatment and allergen avoidance, this treatment method opens a new innovative therapy strategy on the immunomodulatory level for severe equine asthma. Especially noteworthy is the comparatively low number of inhalations (10 × q48h) and the sustained significant improvement over at least eight weeks for all examined clinical, endoscopic, and cytological parameters [77]. The clinical effectiveness could thus be proven in several clinical field studies. The proof of concept (IIa) was verified [76], and the dose (IIb) was determined [77].

This nanoparticulate immunotherapy is independent of specific allergens [78]. In this way, the difficulty of identifying clinically relevant allergens in cases of severe equine asthma can be avoided. This difficulty lies in the multifactorial genesis of severe equine asthma, the existence of different phenotypes, and the diagnostic limitations of commercially available allergy tests [104]. However, White et al. [105] recently developed a microarray platform to detect allergen-specific equine IgE in the serum of severe equine asthma-affected horses against a wide range of presumed allergenic proteins. The microarray revealed an abundance of novel pollen, bacteria, mold, and arthropod proteins, which could play a role in the etiology of severe equine asthma [105]. Furthermore, an IgE latex protein antibody was identified, which showed an association with severe equine asthma-affected horses [105], as this protein is ubiquitous to the horses’ environments in riding surfaces and race tracks [105]. This could potentially open new perspectives in future diagnosis and therapy. As anticipated and in agreement with previous studies [72], the pure GNP application (as a placebo control) showed no clinical or immunological effect in the examined parameters [75,76]. All of the patients involved in the field studies remained in their customary housing conditions (home stables) and were examined without any change in their customary bedding, feed (hay), and exercise in order to accurately test the inhalative CpG-GNP treatment under natural environmental conditions.

The inhalation interval of two days was selected due to the known half-life of CpG-ODN in vivo being up to 48 h [106] and therefore has an advantage over the otherwise necessary once to twice daily administration of inhalative corticosteroids and bronchodilators [2,4]. In order to ensure a safe application of this formulation, possible local and systemic inflammatory reactions or side effects were documented with the help of a modified scoring system according to the Veterinary Cooperative Oncology Group-Common Terminology Criteria of Adverse Events (VCOG-CTCAE) V1.0 [73,75,76]. None of the studies revealed any local (e.g., irritation, redness, follicular hyperplasia, increased mucus production, and cough) or systemic signs of inflammation (e.g., fever, including fibrogen tests and differential blood counts) or any other side effects as a result of the CpG-GNP inhalation treatment. The inhalation enables noninvasive administration with the lowest possible stress and physical impact on the animal and, in comparison to other systemic administration routes (e.g., intralymphatic and subcutaneous [89,102]), has a direct effect on the local pulmonary immune system as well as fewer systemic interactions and thus proves itself to be an ongoing, target-organ-specific, and promising therapeutic approach.

The partially varying initial values of the groups occurred despite random assignment [76,78] and stratified randomization [77]. Due to the heterogenous groups of horses (different stables, stages, and durations of the disease), the character of the study as a field study and the comparatively low number of horses in the individual groups, especially in the placebo group (this, due to the animal welfare aspect, was unavoidable), the starting points of the horses could not be identical. In the placebo group, the differences are especially apparent in the initial values of nasal discharge, breathing type, and the quantity and viscosity of tracheal mucus (four of the ten parameters examined), and in the allergen group, differences are most apparent in neutrophils and viscosity, which were lower than in the other groups, and made the comparison between the groups difficult. The tracheobronchial neutrophil percentage does not correlate well in all cases with the BAL neutrophil percentage, which makes the comparison of some of the results with other studies difficult and does not necessarily allow a direct conclusion about the neutrophil percentage in the lungs. Recently, however, a study comparing tracheal wash and BAL samples in 145 horses, together with endoscopy and mucus findings, found that only 17.5% of horses would have been classified differently if the other method would have been used (reviewed by [10,107]).

The direct comparison of the inhalative beclomethasone application in the study [77] is also problematic, since although the total number of inhalations (10×) is identical to the CpG-GNP application, the interval is different (once daily for the beclomethasone group vs. every second day for the CpG-GNP group) due to their different effect durations. This creates a differing duration of administration (10 days vs. 20 days) and allows no direct comparison between inhalative beclomethasone and CpG-GNP. In addition, cortisone inhalation is typically recommended to be administered twice daily due to the short effect duration, but in practice, this is often not feasible, including in the context of this study due to organizational difficulties (horses were located in their home stables and inhalations were performed by the veterinarians).

In order to observe the influence of the different numbers of inhalations (5, 7, or 10×) with CpG-GNP and to assess an ongoing effect, the effect sizes (Cohen’s d) of all three studies [76,77,78] were calculated and compared for the individual parameters (Table 1). The comparison was made between the initial values before treatment and after four weeks with five inhalation treatments [76], after six weeks with seven inhalations [78], and after eight weeks with ten inhalations [77]. With the exception of the parameters of auscultation and tracheal mucus, the effect sizes for all examined parameters were higher after ten inhalation treatments in comparison to seven or five inhalations. All examined parameters, except for interpleural pressure and clinical scoring, showed a significant clinical effect, d > 0.8 (large clinical effect), while seven parameters were d > 1 (Table 1).

Limiting factors of the inhalative nanoparticulate CpG studies in severe equine asthma-affected horses include the comparatively low numbers of horses per group, as mentioned above. Other factors are the heterogeneity of the groups already referenced (different ages, breeds, uses, seasonal variance in symptom occurrence, genetics, duration of the disease, etc.) and various external influencing factors and housing conditions (influence of various allergen factors) that could not be avoided. With the exception of one study [76], all the others mentioned were performed without a placebo control, which reduces their reliability, and improvements could potentially be attributed to other factors. However, the absence of a placebo control was due to animal welfare concerns and was in accordance with the strict regulations for animal studies. In addition, the subjects used were client-owned horses. The other studies [77,78] compared different therapeutic concepts. Despite randomization, the groups are not exactly homogenous due to the small number of subjects and individual differences, leading to partially varying starting points of the horses within and between groups. However, according to the authors’ opinion, the inhalative CpG immunotherapy shows, at this point, its potential to effect clinically significant improvements, even under these diverse influencing factors in the field. Finally, as there have been no comparable studies on this specific subject by other groups, it has not been possible to provide independent replication, so a risk of author bias in the available data cannot be excluded.

The use of additional allergens after an allergy test as a hyposensitization did not show any significant improvement in comparison to the monotherapy [78]. Because of the difficulty in determining clinically relevant allergens via allergy tests in horses with severe equine asthma due to the disease’s multifactorial nature [104], the authors consider an allergen-independent immunotherapy with CpG-GNP to be much more promising. Human medicine has also seen successful approaches to allergen-independent immunotherapy with CpG and VLPs [108].

The decrease in the quantity and viscosity of secretion in the respiratory tract was one of the most noticeable effects of the CpG-GNP treatment [75,76]. The reduction in neutrophils in the airways, as one of the most important pathomechanisms of the disease, is also a decisive therapeutic effect of the immunotherapy [75,76,77]. That these changes occurred in many horses despite sustained allergen contact is of particular significance, especially considering that corticosteroid treatment often has only mild to no benefit for controlling the neutrophilic inflammation in equine asthma (reviewed by [38,40,47,48]). Interestingly, a recent randomized, double-blind, controlled pilot clinical trial by Mahalingam-Dhingra et al. [109] with lidocaine inhalation treatment (1 mg/kg q12h, 14 d) in severe equine asthma-affected horses showed a significant decrease in bronchoalveolar lavage neutrophil percentage and tracheal mucus score. Both lidocaine and budesonide groups (positive control) had significant decreases in clinical scores [109]. Lidocaine may therefore be an effective and safe treatment for severe equine asthma in horses that cannot tolerate treatment with corticosteroids [109].

The improvements in the partial oxygen pressure at rest and the alveolar–arterial oxygen gradient as well as the interpleural pressure indicate improved ventilation of the lungs [75,76]. This is also reflected in the significantly improved breathing type and the reduction in active expiratory effort [75,76]. This is likely due to a reduction in the secretion in the lumen as well as a decrease in cholinergic bronchospasm [75,76].

As the authors already demonstrated in vivo in equine BAL cells, the CpG-GNP treatment stimulates the upregulation of IL-10 [72,73,74]. This cytokine, produced by Treg cells and others, has a regulatory effect on excessive proinflammatory Th1 and proallergic Th2 immune responses as they occur in the lungs of horses with severe equine asthma. We hypothesize that the modulatory effect of this immunotherapy on inflammatory and allergic reactions mediated by the activation of TLR9 receptors in the lungs will stimulate Treg cells to regulate the dysfunctional Th1/Th2 balance. A unilateral overstimulation of Th1 or Th2 would lead to an enhanced inflammatory or allergic reaction, which explains the importance of this balance and regulation.

In humans, an increase in IL-10 resulting from the activation of Tregs could be attributed to an antiallergic therapy (e.g., the administration of glucocorticoids or allergen-specific immunotherapy) [84]. Asthma patients display significantly lower numbers of Tregs in the BAL of the affected airways in comparison to similar healthy individuals, which correlates with a loss of peripheral allergen tolerance in asthma patients [110]. Jarnicki et al. [81] showed that the application of CpG-ODN effects a release of IL-12 and IL-10 by dendritic cells, which leads to an IL-12-mediated Th1 induction and an IL-10-mediated Treg induction. It can therefore be concluded for the present studies that CpG-ODN effected an IL-10 release via Tregs and thus possibly led to a peripheral tolerance, which would represent an innovative treatment form for allergic equine diseases [52,64,80,85]. Studies have shown that an increase of IFN-γ in conjunction with IL-10 is effective in the inhibition of asthmatic and allergic human diseases (reviewed by [111]).

The significance of IL-10 lies in its inhibitory effect on proallergic IL-4 and IL-5 as well as proinflammatory IFN-γ [82,83]. CpG-ODN also activate the release of IL-10 by B lymphocytes, which regulates and limits CpG-mediated proinflammatory reactions [112]. In humans, CpG-ODN activate plasmacytoid dendritic cells [80]. It has been demonstrated that plasmacytoid dendritic cells (pDCs) form a close-knit network within the human airway epithelia of the upper and lower respiratory tract [113]. Assuming similar conditions in equine lungs, pDCs could also function as the first line of defense and a mediator between the external world and the innate immune system in horses. Moseman et al. [80] demonstrated that CpG-ODN A 2216, via the TLR9 pathway, activates human pDCs to induce CD4^+^CD25^−^ cells in the direction of IL-10-producing CD4^+^CD25^+^ cells in direct cell-to-cell contact. This could also occur in horses in a similar manner, indicating a CpG-ODN-induced Treg activation with IL-10 production. Fewer Tregs are evident in the BAL of asthmatic children than in healthy children [110]. Tregs in the BAL make up 25% of the T-helper cells (in contrast to 5% in PBMCs) [110]. If these results from humans are similarly present in horses, they would explain the elevated IL-10 values in healthy horses after CpG-ODN application via TLR9 activation [74].

The significant downregulation of mRNA expression of CD4^+^ T lymphocytes gained via BAL of Tbet (Th1) and IL-8 (chemotactic to neutrophils) 8 weeks after CpG-GNP administration in comparison to baseline values demonstrates the immunomodulatory effect of CpG-GNP on CD4^+^ T lymphocytes within the airways of severe equine asthma-affected horses [77].

In contrast to other studies [89,99,100,114] in which CpG have been used, this was the first to apply CpG as a monotherapy, via inhalation, and/or bound to gelatin nanoparticles. The advantage of the gelatin nanoparticles as a drug delivery system as opposed to other systems (e.g., virus-like particles) [89] is their good biological tolerability, biodegradability, and aerosol stability as well as their immunologically inert structure [72,88,92,93,94,115]. The mucosal administration activates the local and systemic humoral and cellular immune responses [71].

According to Montamat et al. [116], CpG-ODN have been recently rediscovered for their immune-tolerance-promoting properties, bringing them back into a prominent position as an immune modulator for the treatment of allergic diseases. It has been shown that the appropriate dosage is essential in promoting immune regulation via the recruitment of pDCs. High doses of CpG-ODN trigger an immune tolerance response that can reverse an established allergic milieu. CpG-ODN have been demonstrated to stimulate IL-10-producing B cells and have shown the capacity to prevent and reverse allergic immune reactions in several animal models, indicating their potential as a preventive and active treatment for allergic disease.

## 2. Conclusions

Immunotherapy with CpG-GNP, independent of the causal antigens that may vary geographically and with different endo- and phenotypes, is an effective, allergen-independent, inhalative immunomodulatory therapy with a demonstrated clinical and immunological ongoing effect over at least eight weeks in horses that have suffered for years from severe equine asthma and are often otherwise resistant to conventional therapy. The presently proposed immunotherapy thus opens new perspectives beyond the conventional therapy consisting of symptomatic treatment with corticosteroids and bronchodilators and the often difficult-to-achieve avoidance of antigens. This could also be of interest for human asthma treatment.

## 3. Patents

The authors are co-proprietors of patents EP2399608B1 and US9504760B2.

## Figures and Tables

**Table 1:** Comparison of effect sizes of different parameters after 5, 7, and 10 inhalations of CpG-GNP. In order to evaluate the impact of the number of inhalations with CpG-GNP and to determine the long-term effect after a period of no treatment, the effect sizes (Cohen’s d) of the three different studies were calculated for each individual parameter to facilitate comparisons between different numbers of inhalations and different periods of re-evaluation. The comparisons were performed between the starting point before treatment and after four weeks with five inhalation treatments [76], after six weeks with seven treatments [78], and after eight weeks with ten treatments [77]. With the exception of auscultation and tracheal mucus, the effect sizes for all examined parameters were higher with ten inhalation treatments in comparison to seven or five treatments (Cohen’s *d*: *d* > 0.8: large clinical effect; 0.5–0.8: medium effect; 0.2–0.5: small effect).

Parameters	Effect Size 5 Inhalations	Effect Size 7 Inhalations	Effect Size 10 Inhalations
HOARSI	-	-	2.135
Nasal discharge	1.268	0.764	1.787
Clinical scoring	-	-	0.757
Breathing type	1.333	1.183	1.670
Nasal flaring	-	-	1.538
Respiratory rate	0.341	1.025	1.334
Viscosity	1.177	0.615	1.230
Neutrophils	0.408	0.119	1.064
Auscultation	1.436	1.233	0.973
Tracheal mucus	1.456	0.720	0.964
PaO_2_	0.224	0.739	0.856
AaDO_2_	0.389	0.588	0.829
Interpleural pressure	0.291	0.337	0.551

## Data Availability

The data that support the findings of this review are available from the corresponding author upon reasonable request.

## Abbreviations

AaDO_2_, alveolar–arterial oxygen gradient/arterio-alveolar oxygen pressure difference; APC, antigen-presenting cells; ASIT, allergen-specific immunotherapy; bpm, breaths per minute; BAL, bronchoalveolar lavage; COB, chronic obstructive bronchitis/bronchiolitis; COPD, chronic obstructive pulmonary disease; CpG-GNP, cytosine–phosphate–guanosine gelatin nanoparticles; CpG-ODN, cytosine–phosphate–guanosine oligodeoxynucleotides; DC, dendritic cells; GNP, gelatin nanoparticle; HOARSI, Horse Owner Assigned Respiratory Sign Index; RAO, recurrent airway obstruction; TBS, tracheobronchial secretion; IAD, inflammatory airway disease; IBH, insect bite hypersensitivity; IL, interleukin; IRAK, IL-1 receptor-associated kinase; IFN, interferon; MAPK, mitogen-activated protein kinase; MMP, matrix metalloproteinase; MPLA, monophosphoryl lipid A; MyD88; myeloid differentiation primary response gene 88; NF-κB, nuclear factor-κB; ODN, oligodeoxynucleotide; PAMP, pathogen-associated molecular pattern; PaO_2_ and PaCO_2_, partial oxygen pressure and partial carbon dioxide pressure; PBMCs, peripheral blood mononuclear cells; pDCs, plasmacytoid dendritic cells; Th, T-helper cell; TIMPs, tissue inhibitors of metalloproteinases; TLR-9, toll-like receptor 9; TRAF 6, TNF receptor-associated factor 6; Tregs, T-regulatory cells.
